# The Effect of Combined General Anesthesia and Epidural Analgesia on Postoperative Pulmonary Complications in Thoracoscopic Esophagectomy

**DOI:** 10.3390/medsci14010007

**Published:** 2025-12-23

**Authors:** Hiroyuki Kitagawa, Keiichiro Yokota, Kento Shinnou, Kohei Araki, Norihiro Nishiyama, Hiromichi Maeda, Tsutomu Namikawa, Satoru Seo

**Affiliations:** 1 Department of Surgery, Kochi Medical School, Kohasu, Okocho, Nankoku 783-8505, Kochi, Japan; k.yokota@kochi-u.ac.jp (K.Y.); jm-kentoshinno@kochi-u.ac.jp (K.S.); jm-karaki@kochi-u.ac.jp (K.A.); jm-norinishiyama@kochi-u.ac.jp (N.N.); hmaeda@kochi-u.ac.jp (H.M.); rutosa@kochi-u.ac.jp (S.S.); 2 Department of Clinical Nursing, Kochi Medical School, Kohasu, Okocho, Nankoku 783-8505, Kochi, Japan; tsutomun@kochi-u.ac.jp

**Keywords:** thoracoscopic esophagectomy, epidural analgesia, pulmonary complication, esophageal cancer

## Abstract

**Background and Aims:** Although combined general anesthesia and epidural analgesia are used in open surgery to promote rehabilitation and expectoration, as well as to prevent postoperative pulmonary complications, their effect in thoracoscopic esophagectomy remains unclear. This study aimed to address this issue. **Patients and Methods:** We enrolled 150 patients who underwent thoracoscopic esophagectomy between May 2017 and July 2025. Patient characteristics and postoperative outcomes, including maximum numerical rating scale (NRS) after surgery and pneumonia, were compared between the use and non-use of epidural analgesia. Epidural analgesia was not administered in patients using antithrombotic/anticoagulant drugs or in those with a history of thoracic spine surgery. Postoperative analgesia involved the scheduled administration of acetaminophen in all cases, with patient-controlled analgesia using opioids administered to the non-epidural analgesia group. **Results:** Epidural analgesia was administered to 113 patients (75.3%). The most common levels of epidural catheter placement were Th8/Th9 in 55 patients (36.7%) and Th7/Th8 in 41 patients (27.3%). Laparoscopy was performed in 129 patients (86.0%). Median NRS was five, and pneumonia occurred in 16 patients (10.7%). The epidural anesthesia group had a higher proportion of squamous cell carcinoma (88.5% vs. 73.0%, *p* = 0.024), lower lymphocyte counts (1680 vs. 2065, *p* = 0.020), diabetes (16.8% vs. 37.8%, *p* = 0.007), and hypertension (54.9% vs. 81.1%, *p* = 0.006), and circular stapler anastomosis (83.2% vs. 62.2%, *p* < 0.001). No significant differences were observed in the postoperative NRS, pneumonia, or length of postoperative hospital stay. **Conclusions:** There was no significant difference in the postoperative NRS and pneumonia between those with or without epidural analgesia in thoracoscopic esophagectomy.

## 1. Introduction

Esophagectomy is the cornerstone of curative treatment for esophageal cancer. However, complications are frequent owing to surgical invasion extending into the thoracic and abdominal regions [1], and postoperative pneumonia, in particular, has been reported to worsen the prognosis [2,3]. In this regard, various perioperative management strategies using a multidisciplinary team approach are recommended to reduce the postoperative complications of esophageal cancer [4].

Among these, combined general anesthesia and epidural analgesia during esophagectomy with open thoracotomy and laparotomy is performed to promote rehabilitation and expectoration through postoperative analgesia and to prevent postoperative pneumonia [5]. However, in recent years, minimally invasive thoracoscopic esophagectomy has become widespread, with over half of esophagectomies in Japan now performed thoracoscopically [6]. The efficacy of combined general anesthesia and epidural analgesia in reducing postoperative complications during thoracoscopic esophagectomy remains unclear.

This study aimed to clarify the effect of combining general anesthesia with epidural analgesia in reducing postoperative complications during thoracoscopic esophagectomy for esophageal cancer. The primary endpoint was postoperative pneumonia, and the secondary endpoint was other postoperative complications and length of hospital stay.

## 2. Patients and Methods

This study included 150 patients who underwent thoracoscopic esophagectomy for esophageal cancer between May 2017 and July 2025. Epidural analgesia was not performed in patients using antithrombotic or anticoagulant drugs, or in those with a history of thoracic spine surgery. The placement site of the epidural catheter was determined at the discretion of the anesthesiologist. After epidural catheter placement, the anesthesiologist administered general anesthesia with continuous intravenous infusion of remifentanil at 0.25 μg/kg/min and continuous epidural infusion of 0.25% levobupivacaine for analgesia. The surgery involved right thoracoscopy in the prone position with intrathoracic lymph node dissection, laparoscopic gastric mobilization in the supine position with lymph node dissection, reconstructive gastric tube creation, pulling up to the neck, and cervical anastomosis. Postoperatively, the patient was admitted to the intensive care unit for mechanical ventilation. Extubation was performed the day after surgery, and rehabilitation was initiated. Postoperative analgesia consisted of continuous epidural anesthesia and scheduled intravenous acetaminophen (1000 mg every 8 h) for 5 days [7]. Opioid patient-controlled analgesia was administered as non-epidural anesthesia.

Evaluation parameters included patient background (surgery year periods, sex, age, body mass index, underlying disease, serum albumin level, lymphocyte count, C-reactive protein level, esophageal cancer stage (TNM 8th) [8], and preoperative treatment), surgical variables (mediastinoscopy use [9], laparoscopic manipulation, reconstruction route, reconstructed organ, and anastomosis method), time from entering the operating room to starting surgery and after-hours surgery (time exceeding regular working hours), severity of postoperative pain assessed using the maximum numerical rating scale (NRS) over the first 3 days after surgery, and postoperative complications based on Clavien–Dindo classification [10]. Grade 2 or higher (recurrent laryngeal nerve palsy, pneumonia, anastomotic leakage, wound infection, distant infection, urinary tract infection, and catheter-related infection), and postoperative length of stay, were retrospectively examined. They were classified and compared based on the use or non-use of epidural anesthesia. Pneumonia was defined as a case with symptoms such as fever, cough, and sputum, with infiltration shadows on chest X-Ray or CT, and blood test findings such as elevated white blood cells and CRP levels, and antimicrobial treatment.

Pearson’s chi-squared test was used for categorical variables, and the Mann–Whitney U test was used to evaluate differences in continuous variables. All analyses were performed using JMP 13 (SAS Institute Inc., Cary, NC, USA), with statistical significance set at *p*  <  0.05. The study was approved by the Institutional Review Board of Kochi Medical School (ERB-111984).

## 3. Results

Table 1 shows patient characteristics and comparisons based on the presence or absence of epidural analgesia. The median age was 71 years, and 125 patients (83.3%) were male. Forty-eight percent of all patients had Stage III or IV (advanced) cancer, and 46% received preoperative chemotherapy. The most common underlying diseases were hypertension (61.3%), chronic obstructive pulmonary disease (32.7%), cardiovascular disease (28.0%), and diabetes (22.0%). Robot-assisted surgery was performed in 66 patients (44.0%), hybrid combined mediastinoscopy and right thoracoscopy in 53 (35.3%), and laparoscopic gastric mobilization for abdominal procedures in 129 (86.0%). Epidural anesthesia was administered to 113 (75.3%) patients. The time to the start of surgery, operative time, and after-hours surgery time were 89 min, 575 min, and 146 min, respectively. Median postoperative maximum NRS was five, and pneumonia occurred in 16 patients (10.7%) (Table 2).

Table 3 shows a comparison of outcomes between the use and non-use of intraoperative epidural anesthesia. When categorized by surgical year into early (2017–2021, n = 70) and late (2022–2025, n = 80) periods, epidural analgesia was administered to 58 of 70 cases (82.9%) in the early group and 55 of 80 cases (68.8%) in the late group (*p* = 0.046). There was no significant difference in age at the period (Early group 70 years vs. Late group 71 years, *p* = 0.210) or anticoagulant use (Early group 12.9% vs. Late group 17.5%, *p* = 0.431). The levels of epidural catheter insertion were Th8/Th9 in 55 patients (36.7%) and Th7/Th8 in 41 patients (27.3%). No hematomas or infectious complications associated with the epidural catheter were observed.

Those who received epidural analgesia showed a higher proportion of squamous cell carcinoma histology (88.5% vs. 73.0%, *p* = 0.024) and higher lymphocyte counts in blood tests (1680/μL vs. 2065/μL, *p* = 0.020) than those who did not receive epidural analgesia. Comorbidities were more common in the non-epidural anesthesia group, with statistically significant differences in the prevalence of diabetes (16.8% vs. 37.8%, *p* = 0.007) and hypertension (54.9% vs. 81.1%, *p* = 0.006). The reconstruction route was more frequently retrosternal in the epidural analgesia group and posterior mediastinal in the non-epidural anesthesia group. Circular stapler anastomosis was more frequently used (83.2% vs. 62.2%) in the epidural analgesia group, whereas other anastomosis methods were more common in the non-epidural analgesia group. Preoperative treatment was more frequent in the epidural analgesia group, although the difference was not significant.

No significant differences were observed between the two groups in terms of the proportion of laparoscopic procedures, operative time, blood loss, intraoperative fluid administration, maximum NRS, postoperative complication rates, or length of postoperative hospital stay. After excluding the use of antithrombotic or anticoagulant drugs, no significant difference in the incidence of postoperative pneumonia was observed between the epidural analgesia group and the non-epidural analgesia group (10.9% vs. 0.0%, *p* = 0.152). Robot-assisted surgery was slightly more common in the group without epidural analgesia (39.8% vs. 56.8%; *p* = 0.072); however, no significant difference was observed between the presence or absence of robot-assisted surgery and NRS (5 vs. 5; *p* = 0.846) or the incidence of postoperative pneumonia (12.1% vs. 9.5%; *p* = 0.608). The group with an epidural catheter placed preoperatively on the day of surgery trended to have a longer time to the start of surgery (84 min vs. 92 min, *p* = 0.111) and after-hours surgery (111 min vs. 155 min, *p* = 0.095). There was no significant difference between the epidural catheter placement level and postoperative pain scores, and postoperative pneumonia (Table 4).

Patients with postoperative pneumonia had a higher prevalence of diabetes compared to those without pneumonia (50.0% vs. 18.7%, *p* = 0.004) and a lower rate of NAC (12.5% vs. 50.0%, *p* = 0.004). In multivariate logistic analysis, patients with diabetes (odds ratio: 4.217; 95% confidence interval (CI): 1.384–12.843; *p* = 0.011) were significant risk factors for pneumonia, and neoadjuvant chemotherapy (NAC) tended to reduce the risk (odds ratio: 0.147; 95% CI: 0.031–0.685; *p* = 0.015).

## 4. Discussion

In conventional open gastrointestinal surgery, the combination of general and epidural anesthesia has been considered to promote postoperative recovery by alleviating postoperative pain and suppressing protein catabolism [11]. In esophagectomy, reports indicate that the combination of general and epidural anesthesia not only provides postoperative analgesia but also suppresses the elevation in inflammatory stress hormones [12]. Meta-analyses of esophagectomy have reported that epidural analgesia not only aids respiratory recovery and shortens intensive care unit stay through postoperative analgesia [13] but also reduces pulmonary complications [14]. However, this may have been influenced by the shift from traditional open thoracotomy and laparotomy to minimally invasive surgery via thoracoscopy and laparoscopy [15].

It was previously reported that inserting an epidural catheter at the Th7/Th8 level significantly reduced postoperative pain compared to placement over Th6/Th7 or Th8/Th9 during esophagectomy. However, in thoracoscopic surgery combined with laparoscopic surgery, epidural catheter placement under Th8/Th9 significantly increases postoperative pain [16]. This suggests that in esophagectomy involving both thoracic and abdominal procedures, the effect of epidural analgesia on reducing postoperative pulmonary complications may be limited in thoracoscopic surgery compared with open surgery. Therefore, a PubMed search using the keywords [thoracoscopic esophagectomy] and [epidural anesthesia] yielded the results shown in Table 5 [17,18,19,20]. Studies reported that inserting an epidural catheter at a level higher than Th7 resulted in a reduction in pulmonary complications compared to intravenous analgesia [18], whereas studies reported that inserting it below Th7 showed no reduction in pulmonary complications [17,20].

A randomized controlled trial comparing thoracoscopic and open surgery for esophagectomy demonstrated a reduction in pulmonary complications with thoracoscopic surgery [21]. Conversely, the ROMIO randomized clinical trial comparing open surgery with laparoscopic or open abdominal procedures found no difference in postoperative complication rates between the open and laparoscopic approaches [22]. A real-world study using the Japanese national clinical database also compared abdominal procedures during thoracoscopic esophagectomy between the open and laparoscopic approaches and found no significant differences in pulmonary complication rates [23]. These findings suggest that minimizing thoracic wall damage via thoracoscopy and providing thoracic analgesia through high-level epidural anesthesia are more important than abdominal analgesia for reducing pulmonary complications. In this study, 46 of the 113 patients who received epidural analgesia had a catheter placement of Th7/Th8 or above, and 67 (59.3%) had Th8/Th9 or below; however, there was no significant association between epidural catheter placement level and postoperative pain scores, and postoperative pneumonia. We initiated open thoracotomy and total laparoscopic gastric mobilization in 2005 [24], switching to combined thoracoscopic and laparoscopic techniques in 2009. However, because we performed a small laparotomy in the epigastrium to confirm blood flow in the reconstructed gastric conduit using near-infrared fluorescence after laparoscopic gastric mobilization [25], the difference in postoperative pain between the open and laparoscopic approaches may have been reduced. The use of additional analgesic methods, including scheduled intravenous acetaminophen [7], may have influenced the incidence of postoperative pulmonary complications based on the use or non-use of epidural analgesia. Moreover, the aging patient population has increased the prevalence of concomitant atrial fibrillation, leading to an increasing trend in patients using thromboembolic prophylaxis and anticoagulants [26]. Since epidural anesthesia is contraindicated in these patients, diverse analgesia methods are crucial, such as performing wound infiltration anesthesia by injecting a local anesthetic into the surgical site before surgery concludes [27]. Furthermore, if after-hours surgery can be reduced by omitting epidural catheter insertion, it may lead to a reduction in labor costs.

The limitations of this study include the biases inherent to retrospective, small-sample, comparative research. For example, the non-epidural analgesia group had a higher prevalence of underlying comorbidities, such as hypertension and diabetes, use of antithrombotic or anticoagulant drugs, a higher proportion of robotic surgeries, and higher lymphocyte counts. These factors may have influenced surgical invasiveness and patient immunity, and these patients inherently have a higher perioperative risk. Also, time-related heterogeneity about advance of the surgical techniques, perioperative care, enhanced recovery protocols, and anesthesia practice might affect the postoperative complications.

In addition, the placement site of the epidural catheter and the physicians performing the catheter insertion were not standardized. Due to the limited number of subgroup samples at the epidural catheter level, statistical power was insufficient. Furthermore, while the protocol of the postoperative opioid administration was established, the quantified dosage (total mg) used by each patient was not retrospectively available. Data were also unavailable regarding the use of rescue analgesics, the incidence of postoperative nausea and vomiting, and the duration of ileus.

## 5. Conclusions

In this study, there was no significant difference in the complication rate of postoperative pneumonia with or without epidural analgesia during thoracoscopic esophagectomy. Omitting epidural analgesia may lead to more efficient utilization of medical resources and the length of time involved in esophagectomy; however, further accumulation of cases and investigations is necessary to reveal this.

## Figures and Tables

**Table 1:** Characteristics of patients who underwent thoracoscopic esophagectomy.

	n = 150
Surgery year periods	
Early (2017–2021) (%)	70 (46.7)
Late (2022–2025) (%)	80 (53.3)
Age, years	71 (36–91)
Sex, male (%)	125 (83.3)
Histological type	
Squamous cell carcinoma (%)	127 (84.7)
Adenocarcinoma (%)	15 (10.0)
Other type (%)	8 (5.3)
Tumor location	
Ce/Ut (%)	38 (25.3)
Mt/Lt/Jz (%)	112 (74.7)
Clinical stage	
Ⅰ (%)	58 (38.7)
Ⅱ (%)	20 (13.3)
Ⅲ (%)	35 (23.3)
ⅣA (%)	13 (8.7)
ⅣB (%)	24 (16.0)
Body mass index (kg/m^2^)	22.2 (14.0–40.2)
Albumin (g/dL)	4.0 (1.5–5.1)
Lymphocyte (/μL)	1760 (320–9560)
C-reactive protein (g/dL)	0.1 (0.0–4.1)
Comorbidities	
Diabetes (%)	33 (22.0)
Cardiovascular disease (%)	42 (28.0)
Hypertension (%)	92 (61.3)
COPD (%)	49 (32.7)
Antithrombotic or anticoagulant drugs (%)	23 (15.3)
Steroid use (%)	9 (6.0)
Previous radiation therapy (%)	20 (13.3)
Neoadjuvant chemotherapy (%)	69 (46.0)
Salvage surgery (%)	11 (7.3)
Surgical procedure	
Robot-assisted (%)	66 (44.0)
Hybrid with mediastinoscopy (%)	53 (35.3)
Laparoscopic (%)	129 (86.0)
Supraclavicular dissection (%)	65 (43.3)
Reconstruction route	
Post-mediastinal (%)	90 (60.0)
Retrosternal (%)	59 (39.3)
Ante-sternal (%)	1 (0.7)
Reconstructive substitute	
Stomach (%)	145 (96.7)
Colon (%)	5 (3.3)
Anastomosis method	
Circular stapler (%)	117 (78.0)
Modified-Collard (%)	15 (10.0)
Triangle (%)	8 (5.3)
Handsewn (%)	10 (6.7)
Feeding catheter	
Gastro-duodenum (%)	108 (72.0)
Jejunum (%)	42 (28.0)

Categorical variables are expressed as n (%), and continuous variables are expressed as median (range). Ce—cervical esophagus; Ut—upper thoracic; Mt—middle thoracic; Lt—lower thoracic; Jz—junctional zone; COPD—chronic obstructive pulmonary disease.

**Table 2:** Outcomes of patients who underwent thoracoscopic esophagectomy.

Surgical Outcomes	n = 150
Time to start surgery (min)	89 (13–167)
Operative time, min	575 (379–748)
Thoracoscopic time, min	214 (78–385)
Fluid infusion, mL	3750 (2190–6300)
Blood loss, mL	125 (0–950)
After-hours surgery (min)	146 (−58–397)
Maximum numerical rating scale	5 (0–10)
Postoperative complications	
Recurrent laryngeal nerve palsy (%)	45 (30.0)
Pneumonia (%)	16 (10.7)
Anastomotic leakage (%)	26 (17.3)
Wound infection (%)	48 (32.0)
Distant infections (%)	15 (10.0)
Hospital stays (days)	26 (10–164)

**Table 3:** Comparison of outcomes between the use and non-use of intraoperative epidural anesthesia.

	Epidural Analgesia		*p*-Value
	Yes (n = 113)	No (n = 37)	
Surgery year period			0.046
Early (2017–2021) (%)	58 (51.3)	25 (67.6)	
Late (2022–2025) (%)	55 (48.7)	12 (32.4)	
Age, years	71 (36–91)	70 (49–84)	0.333
Sex, male (%)	92 (81.4)	33 (89.2)	0.271
Histological type			0.024
Squamous cell carcinoma (%)	100 (88.5)	27 (73.0)	
Adenocarcinoma (%)	7 (6.2)	8 (21.6)	
Other type (%)	6 (5.3)	2 (5.4)	
Tumor location			0.785
Ce/Ut (%)	28 (24.8)	10 (27.0)	
Mt/Lt/Jz (%)	85 (75.2)	27 (73.0)	
Clinical stage			0.080
Ⅰ (%)	43 (38.1)	15 (40.5)	
Ⅱ (%)	16 (14.2)	4 (10.8)	
Ⅲ (%)	27 (23.9)	8 (21.6)	
ⅣA (%)	13 (11.5)	0 (0.0)	
ⅣB (%)	14 (12.4)	10 (27.0)	
Body mass index (kg/m^2^)	22.0 (14.0–33.1)	22.5 (18.1–40.2)	0.097
Albumin (g/dL)	4.0 (1.5–4.6)	4.0 (2.8–5.1)	0.768
Lymphocyte (/μL)	1680 (320–9560)	2065 (560–5690)	0.020
C-reactive protein (g/dL)	0.2 (0.0–2.7)	0.1 (0.0–4.1)	0.747
Comorbidities			
Diabetes (%)	19 (16.8)	14 (37.8)	0.007
Cardiovascular disease (%)	28 (24.8)	14 (37.8)	0.125
Hypertension (%)	62 (54.9)	30 (81.1)	0.006
COPD (%)	33 (29.2)	16 (43.2)	0.114
Antithrombotic or anticoagulant drugs (%)	3 (2.7)	20 (54.1)	<0.001
Steroid use (%)	5 (4.4)	4 (10.8)	0.156
Previous radiation therapy (%)	16 (14.2)	4 (10.8)	0.603
Neoadjuvant chemotherapy (%)	55 (48.7)	14 (37.8)	0.251
Salvage surgery (%)	8 (7.1)	3 (8.1)	0.835
Surgical procedure			
Robot-assisted (%)	45 (39.8)	21 (56.8)	0.072
Hybrid with mediastinoscopy (%)	44 (38.9)	9 (24.3)	0.107
Laparoscopic (%)	95 (84.1)	34 (92.0)	0.234
Supraclavicular dissection (%)	50 (44.3)	15 (40.5)	0.207
Reconstruction route			0.098
Post-mediastinal (%)	73 (64.6)	17 (46.0)	
Retrosternal (%)	39 (34.5)	20 (54.1)	
Ante-sternal (%)	1 (0.9)	0 (0.0)	
Reconstructive substitute			0.806
Stomach (%)	109 (96.5)	36 (97.3)	
Colon (%)	4 (3.5)	1 (2.7)	
Anastomosis method			<0.001
Circular stapler (%)	94 (83.2)	23 (62.2)	
Modified-Collard (%)	12 (10.6)	3 (8.1)	
Triangle (%)	1 (0.9)	7 (18.9)	
Handsewn (%)	6 (5.3)	4 (10.8)	
Feeding catheter			0.879
Gastro-duodenum (%)	81 (71.7)	27 (73.0)	
Jejunum (%)	32 (28.3)	10 (27.0)	
Surgical outcomes			
Time to start surgery (min)	92 (13–167)	84 (53–133)	0.111
Operative time, min	582 (379–748)	568 (461–735)	0.467
Thoracoscopic time, min	211 (78–363)	224 (99–385)	0.529
Fluid infusion, mL	3770 (2190–6250)	3750 (2320–6300)	0.603
Blood loss, mL	125 (0–670)	140 (30–950)	0.560
After-hours surgery (min)	155 (−58–397)	111 (16–336)	0.095
Maximum numerical rating scale	4 (0–10)	5 (1–8)	0.365
Postoperative complications			
Recurrent laryngeal nerve palsy (%)	31 (27.4)	14 (37.8)	0.231
Pneumonia (%)	12 (10.6)	4 (10.8)	0.974
Anastomotic leakage (%)	21 (18.6)	5 (13.5)	0.479
Wound infection (%)	35 (31.0)	13 (35.1)	0.638
Distant infections (%)	13 (11.5)	2 (5.4)	0.283
Hospital stays (days)	26 (10–164)	28 (13–97)	0.574

Categorical variables are expressed as n (%), and continuous variables are expressed as median (range). Ce—cervical esophagus; Ut—upper thoracic; Mt—middle thoracic; Lt—lower thoracic; Jz—junctional zone; COPD—chronic obstructive pulmonary disease.

**Table 4:** Correlation between the epidural catheter level and postoperative maximum NRS and pneumonia.

Epidural Catheter Level	n (%)	Maximum NRS	*p*-Value	Pneumonia (%)	*p*-Value
Th6/Th7	5 (4.4)	4 (2–7)	0.604	0 (0.0)	0.744
Th7/Th8	41 (36.3)	5 (0–8)		6 (14.6)	
Th8/Th9	55 (48.7)	4 (0–10)		6 (10.9)	
Th9/Th10	11 (9.7)	5 (3–9)		0 (0.0)	
Th10/Th11	1 (0.9)	5 (3–9)		0 (0.0)	

Th—thoracic vertebrae; NRS—numerical rating scale.

**Table 5:** Previously controlled studies of epidural analgesia during thoracoscopic esophagectomy.

Author, Year	Number of Patients	Abdominal Procedure	Level of Epidural Catheter	Control Group	Outcomes
Wei K, 2018 [17]	28 with continuous injection	Laparoscopic	Th7/Th8	27 with intermittent injection	Postoperative complications were not different between the two groups.
Han X, 2000 [20]	38 with epidural anesthesia	Laparoscopic	Th7/Th8 or Th8/Th9	38 without epidural anesthesia	Better QOL score in the epidural anesthesia group.
She H, 2023 [18]	64 with TPVB	Not described	Th4 and Th7	83 with epidural block	TPVB was better for maintaining intraoperative blood pressure, heart rate stability, and postoperative survival.
Xu M, 2023 [19]	56 with TPVB and subcostal TAP	Not described	Th6 and Th9	56 with PCEA and 56 with PCIA	VAS in the TPVB with PVB group was higher than that in the PCEA group. The pulmonary complication rate in the PCIA group was significantly higher than that in the PCEA group.

QOL—quality of life; TPVB—thoracic paravertebral block; TAP—transverse abdominis plane block; PCEA—patient-controlled epidural analgesia; PCIA—patient-controlled intravenous analgesia.

## Data Availability

The data presented in this study are available on request from the corresponding author due to their containing information that could compromise the privacy of research participants.
